# Effect of intraplaque angiogenesis to atherosclerotic rupture-prone plaque induced by high shear stress in rabbit model

**DOI:** 10.1093/rb/rbx007

**Published:** 2017-07-07

**Authors:** Juhui Qiu, Daoxi Lei, Jianjun Hu, Tieying Yin, Kang Zhang, Donghong Yu, Guixue Wang

**Affiliations:** * Department of Bio-engineering, Key Laboratory for Biorheological Science and Technology of Ministry of Education, State and Local Joint Engineering Laboratory for Vascular Implants, Bioengineering College of Chongqing University, Chongqing 400044, China

**Keywords:** angiogenesis, atherosclerosis, shear stress, prone-rupture plaque

## Abstract

Atherosclerotic prone-rupture plaque is mainly localized in the region of the entrance to the stenosis with high shear stress and the reasons are largely unknown. Our hypothesis is that such a distribution of cells in atherosclerotic plaque may depend on the angiogenesis. Silastic collars induced regions of high shear stress (20.68 ± 5.27 dynes/cm^2^) in the upstream flow and low shear stress (12.25 ± 1.28 dynes/cm^2^) in the downstream flow in carotid arteries. Compared with the low shear stress region, plaques in the high shear stress region showed more intraplaque haemorrhaging, less collagen and higher apoptotic rates of vascular smooth muscle cells; endothelial cells (ECs) in the high shear stress region were characterized with integrity and high endothelial nitric oxide synthase (eNOS) expression (1570.3 ± 345.5% vs 172.9 ± 49.9%). The number of intraplaque microvessels is very high in the high shear stress region (15 ± 1.8 n/mm^2^ vs 3.5 ± 0.4 n/mm^2^), and the microvessels in the plaque show ECs were abnormal, with membrane blebs, intracytoplasmic vacuoles and leukocyte infiltration. Our current study reveals that the integrity of the endothelium and the vulnerability of atherosclerotic plaques are simultaneously localized in high shear stress regions, and we provide evidence for the first time that microvessels in the intraplaque maybe responsible for rupture-prone plaque formation in the high shear stress region.

## Introduction

Coronary artery diseases such as acute coronary syndromes (ACS), are the leading cause of death worldwide [1, 2]. ACS events are frequently caused by rupture of vulnerable plaque and then thrombus formation, which ultimately leads to distal cessation of blood flow. Vulnerable plaque mainly occurs in mildly obstructive coronary lesions rather than severely stenosed (>50%) lesions [3]. Rupture-prone plaques are characterized by their specific morphology and composition: a large lipid core separated from the vessel lumen by a thin fibrous cap and inflammation with higher macrophages counts [4]. It is well-known that local shear stress plays an essential role in plaque formation, progression and rupture. Plaque rupture in the carotid artery is mostly localized at the upstream region of the minimal lumen site with high shear stress confirmed by animal and clinical researches [5–15]. But the reason why atherosclerotic plaques rupture occurs at this region is currently unknown. Segers *et al*. proposed that the accumulation of oxidized low-density lipoprotein (ox-LDL) may lead to the spatially restricted distribution of activated macrophages in plaques *in vivo* [5]. However, high shear stress is well known to be endothelium-protective and the endothelium cells may obstruct the low-density lipoprotein(LDL)from entering into the vessel wall, and also ox-LDL is mainly accumulated within the downstream region with low shear stress [13]. Therefore, there should be some other potentially critical factors stimulate plaques progression and rupture.

Recently, several studies have revealed that plaque angiogenesis in the vessel wall promotes the growth of atheromas and is involved in the process of atherosclerosis plaque rupture, and the new vasa vasorum serves as a conduit into the vessel wall for cellular and soluble components, such as red blood cells (RBCs), inflammatory cells and lipid/lipoproteins [16, 17]. Furthermore, most microvessels were thin-walled in atherosclerotic arteries, and the compromised structural integrity of microvascular endothelium may interpret the association between the microvascular leakage and intraplaque haemorrhage in advanced human coronary atherosclerosis [18]. In addition, high shear stress enhances the expression of vascular endothelial growth factor (VEGF) [19, 20], and the vascular ECs can convey the stimulation from the lumens to the endothelium and then induce angiogenesis [17]. Since angiogenesis may be induced by high shear stress and plays an important role in the accumulation of inflammatory cells and lipid/lipoproteins in the intra-plaque, does it play an important role in the development of atherosclerosis plaque rupture induced by high shear stress?

We proposed that angiogenesis is the reason why rupture-prone plaques is localized in high shear stress region and is related to blood flow in plaque composition and vascular remodeling [21]. Based on the perivascular silastic collars model, we provide evidence that ECs in the high shear stress region were characterized with integrity and high eNOS expression. The number of intraplaque microvessels is very high in the high shear stress region, and the microvessels in the plaque show ECs were abnormal, with membrane blebs, intracytoplasmic vacuoles and leukocyte infiltration.

## Materials and methods

### Experimental animal model

**Figure 1:**
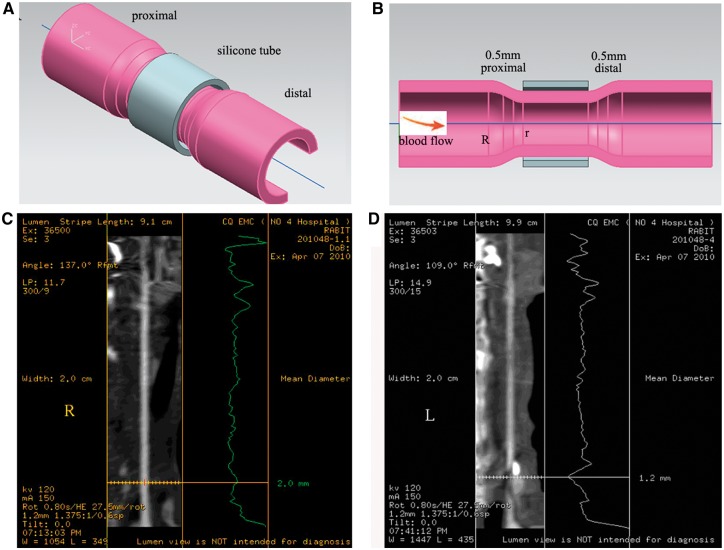
The model of perivascular collar placement. A stenosis of the vessel presents the stenosis of the plaque intrudes into the lumen (**A** and **B**). computed tomography (CT) scanner detects the diameter of the vascular lumen in the control right (**C**) and left (**D**) carotid artery

The Guide for Chinese Animal Care and Use Committee standards was followed for the animal housing and surgical procedures. All procedures were done in accordance with protocols approved by the Animal Ethics Committee of Chongqing University. Collars were prepared from silastic tubing and autoclaved before use (Fig. 1A–D). Animal models were based on our previously published method [22]. Because we previously had built the control animals group (sham operation, non-constrictive cuff placement), this manuscript and its conclusions rely solely on the rabbit constrictive cuff model. Since this is a surgical procedure and no adequate are presented. Twelve young rabbits were subjected to surgical carotid artery ringers for stenosis (the length of the collar was 8 mm), and then divided randomly into two groups, one for histological analysis and the other for electron microscopy analysis. The animals were fed with a Western-type diet (1% cholesterol and 5% lard) for 8 weeks after surgery. Briefly, each rabbit was anaesthetized with pentobarbital sodium (30 mg/kg, 30 mg/ml), fixed and depilated. Signs of adequate anaesthesia were continuously monitored during surgery and were defined as increases of heart rate (HR) by over 20% of the pre-anaesthetic values, or the disappearance of animal movement, coughing, or jerking. Both carotid sheaths were opened, and 2% lidocaine was placed in the wound as a local anaesthetic. The common carotid arteries were then dissected free from the surrounding connective tissue, avoiding damage to the vagus nerves and carotid bodies. In order to quantify the stenosis rate (to control the stenosis more precisely), we took pictures of the vessel after separating the vascular tissue and measured the outside diameter using drawing software. Forty percent stenosis was controlled using the equation below:

r=0.775R

Where *r* represents the inside diameter of the silastic collar and *R* represents the outside diameter of the vessel.

Subsequently, the silastic tube was placed around the left common carotid artery and fixed using operation clew for 3 circumferential silk ties. Finally, the skin of the neck was sewn up and closed.

### Shear stress determination

After implantation of the perivascular carotid collar, blood flows through the upstream and downstream directions of the left carotid artery were measured in six rabbits using a transit-time electromagnetic flowmeter (TS420, Transonic Systems Inc., Ithaca, NY) with a flow probe (2 mm). The probe was mounted on the left common carotid artery from the upstream flow of the stenosis to the downstream for approximately 40 min. Flow rate was recorded on a Gould 2000 chart recorder. Blood flow rates in the experimental right common carotid arteries were measured at the same time. Flow rate measurements were made from the upstream to the downstream directions in the left carotid arteries of six rabbits at random. Dimensions were evaluated by computed tomographic angiography.

Blood flow rates and diameter data were combined to calculate time-averaged shear stress using a Poiseiulle flow approximation:

$$\tau =4\mu Q/\pi r^{3}$$

Where *μ* = 0.035 (the viscosity of blood) [23], *Q* is the blood flow rate, and *r* is the radius of the blood vessel.

### Tissue preparation and histological analysis

Six rabbits were sacrificed at the end of 8 weeks by bloodletting through the arterial cannula using pentobarbital sodium to anesthetize the animal. The tissues were harvested, washed clean with isotonic NaCl, and then fixed in 4% paraformaldehyde. The right control carotid artery was also separated, and the vessel was washed clean as above. The left carotid artery ringer was then divided into two parts, and in each section we obtained 1–5 mm proximal part and distal part to the silastic tube. After the vascular tissues were fixed by paraformaldehyde, dehydrated, and paraffin embedding, slicing, Hematoxylin and eosin (HE) staining, and Masson trichrome staining were carried out as usual. Internal elastic lamina (IEL) was determined by Verhoeff’s van Gieson staining.

### Electron microscopy

The six other rabbits were also sacrificed at the end of 8 weeks following the steps above and tissues were fixed in 2.5% glutaraldehyde. Using small eye scissors, the vessels were slightly split. The harvested tissues were processed for scanning electron microscopy (SEM). Briefly, after fixing, tissue sections were dehydrated in a series of alcohol, critical-point dried and prepared for examination with a scanning electron microscope (TESCAN VEGA2) using standard procedures.

The harvested vessel was processed for transmission electron microscopy (TEM), and postfixed in 1% osmium tetroxide. Thin sections (60–90 nm) were obtained, placed on copper grids, and stained with uranyl acetate and lead citrate. These were examined with a JEOL-100CX II electron microscope (Japanese Electron Optical Laboratories), and representative photographs were taken. TEM studies were mainly used as means of verifying the presence of apoptosis.

### Immunostaining

The TUNEL method was used with minor modifications based on previous research [24]. Specifically, optimal proteolysis required the application of 100 μg/ml proteinase K for 30 min. An apoptosis detection kit was used with chromogen diaminobenzidine (DAB). The counterstain was hematoxylin. Biochemical controls were performed with positive control slides treated with DNase-1 and with staining in the absence of terminal deoxynucleotidyl transferase enzyme as the negative control. The expression of eNOS was immediately detected with eNOS-antibodies on arterial sections.

### Quantification of chemical and immunohistochemistry staining

The images were taken with a microscope (Olympus BX81; Olympus, Tokyo, Japan), and processing of data was implemented with the aid of Image Pro Plus software. Masson trichrome staining was shown as the area percentage of the positive areas (100× magnification). Apoptotic cells showing morphological features characteristic of apoptosis, in addition to positive TUNEL reactions, were considered to be apoptotic. Nonspecific cytoplasmic staining without nuclear involvement was considered negative. (TUNEL labeling index = TUNEL-positive cells/total cells per HPF × 100). Results are expressed as mean ± SD. The expression of eNOS was quantified (100× magnification) and showed the total OD value.

### Statistical analysis

Shear stress levels and chemical and immunohistochemical staining results were expressed as mean ± SD (*n* = 6). Comparisons were performed using the independent-sample *t* test. All statistical work was performed with SPSS 17.0 software.

## Results

### High shear stress localized on the upstream of stenosis

**Figure 2:**
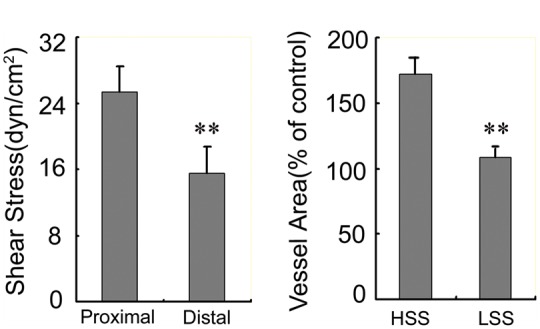
(**A**) High shear stress exists in the upstream direction of the stenosis **P < 0.01, relative to the proximal of the stenosis. (**B**) High shear stress induces vascular expansive remodeling. **P < 0.01, relative to the high shear stress regions

The blood flow rate in the upstream direction of the left carotid artery increased by 38.7% (25.42 ± 3.01 ml/min) compared with the downstream direction of the left common carotid artery (15.58 ± 3.13 ml/min). The vessel diameter showed no obvious change from the upstream to the downstream directions of the left carotid artery. We combined flow rates and diameters from every rabbit to estimate time-averaged shear stress and then averaged it. The shear stress in the upstream direction of the left carotid artery increased by 41.7% (20.68 ± 5.27 dynes/cm^2^) compared with the downstream direction of the left common carotid artery (12.25 ± 1.28 dynes/cm^2^) (Fig. 2A). Therefore, high wall shear stress regions were mainly localized in the upstream direction of the collar with perivascular carotid collar placement.

### Rupture-prone plaques localized on the high shear stress region

Atherosclerotic plaques with excessive expansive remodeling evolve gradually to thin cap fibroatheroma, and further develop into high-risk plaques [25]. To determine whether or not high shear stress can induce vascular expansive remodeling, the vessel area was measured. We found that high shear stress induced expansive remodeling. The vascular cross-sectional area increased in high shear stress regions to 1.8-fold (Fig. 2B) compared with low shear stress regions.

**Figure 3:**
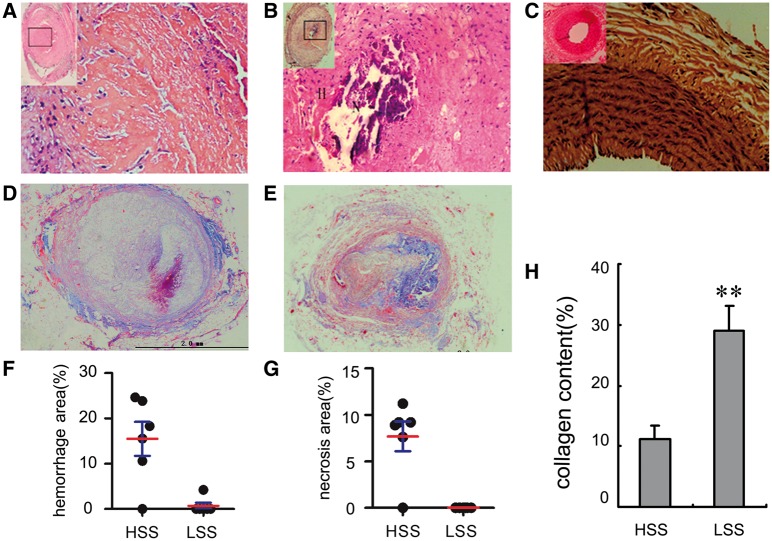
Haemorrhaging and visible necrosis mainly localize in the high shear stress region. (**A**) Haemorrhages may exist without visible necrosis in the high shear region. (**B**) Haemorrhages can also exist with visible necrosis in the upstream direction of the stenosis with high shear stress. (**C**) No haemorrhaging or visible necrosis exists in low shear stress regions. (**F** and **G**) On gross examination haemorrhaging and necrosis. (**D**) Masson straining for collagen (40× magnification) and, almost no collagen is found in the plaque in high shear stress regions, blue color represents collagen. (**E**) Collagen on the intimal hyperplasia is localized in low shear stress regions. (**H**) Quantification of the percentage of areas in the intima positive for collagen and high shear stress administration decreases intimal collagen in atherosclerotic lesions.**P < 0.01, relative to the high shear stress regions

Upon gross examination, haemorrhaging and cellular necrosis were commonly observed in the upstream direction of carotid collar placement (Fig. 3A–C). Gross haemorrhaging and extensive necrosis almost always emerged in high shear stress regions (83%, *n* = 6). In contrast, rare haemorrhaging (16.7%, *n* = 6) and no gross necrosis were seen in low shear stress regions (Fig. 3F and G).

**Figure 4:**
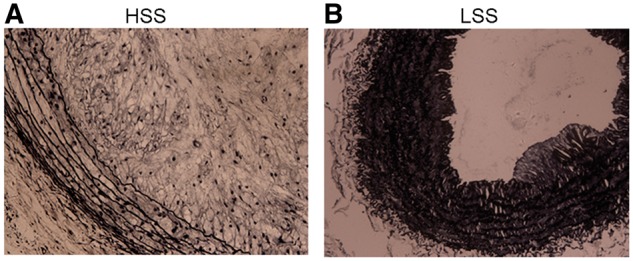
Internal elastic lamina was stretched straight in high shear stress regions. (**A**) IEL in the high shear stress region. (**B**) IEL in the low shear stress region

To conﬁrm plaque characteristics, upstream and downstream plaques were evaluated by Masson trichrome and Verhoeff’s van Gieson staining. Plaques in high shear stress regions showed characteristics of a vulnerable phenotype. The collagen content in these regions was less (10.6 ± 0.5% versus 33.1 ± 1.2%, *P* < 0.01) (Fig. 3D and E) compared with that in low shear stress regions (Fig. 3H). The IEL in high shear stress regions was stretched straight and defects were observed, and this was in comparison with the IEL in low shear stress regions (Fig. 4A and B).

**Figure 5:**
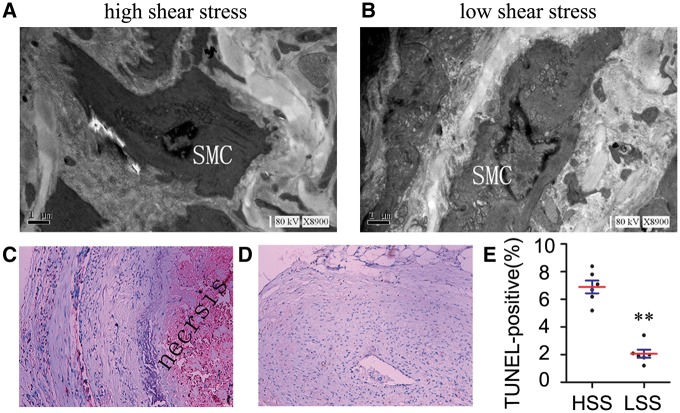
VSMCs have a high apoptotic rate in high shear stress regions. Transmission electron micrographs and immunohistochemical staining with TUNEL demonstrated the apoptosis of VSMCs in the plaque in high and low shear stress regions. (**A**) Electron micrographs from an artery 8 weeks after carotid collar placement showed nuclear and cytoplasmic abnormalities characteristic of apoptotic VSMCs in high shear stress regions. Condensed chromatin forming crescents in apposition to the nuclear envelope is characteristic of the early stages of apoptosis. The dash line represents the condensed chromatin region. (**B**) Cells localized in the downstream direction contain an increasing number of mitochondria, rough endoplasmic reticulum and golgi apparatus in the cell stomata and cytoplasmic processes (8900× magnification). (**C**) Cell apoptosis was detected by TUNEL-staining (100× magnification). (**D**) Fewer apoptotic cells are found in low shear stress regions. (**E**) Quantification of the percentage of positive areas for TUNEL-staining, and high shear stress administration increases apoptosis of the intimal VSMCs in atherosclerotic lesions.** *P* < 0.01, relative to the high shear stress regions

Vascular smooth muscle cells (VSMCs) apoptosis alone is sufficient to induce features of plaque vulnerability in atherosclerosis [26]. To explore the relationship between the apoptotic rate of VSMCs and the shear stress rate, we examined the apoptosis of VSMCs by TEM. VSMCs revealed the characteristics of early-stage apoptosis in high shear stress regions. The ultrastructural features of such identified VSMCs included cell shrinkage, membrane blebbing and chromatin condensation. Nuclei were coagulated, and chromatins were highly condensed and became marginal and vacuolated (Fig. 5A). VSMCs localized in low shear stress regions contained an increasing number of mitochondria, rough endoplasmic reticulum, and Golgi apparatus in the cell stomata and cytoplasmic; these cells had obvious active ‘synthetic’ phenotypes (Fig. 5B). TUNEL staining in the arterial sections with light microscopy showed that high shear stress regions had a high apoptotic rate of VSMCs (7.03 ± 2.22), compared with low shear stress regions (1.87 ± 0.49) (Fig. 5C–E).

### The ECs on the high shear stress region were normal and had normal function

**Figure 6:**
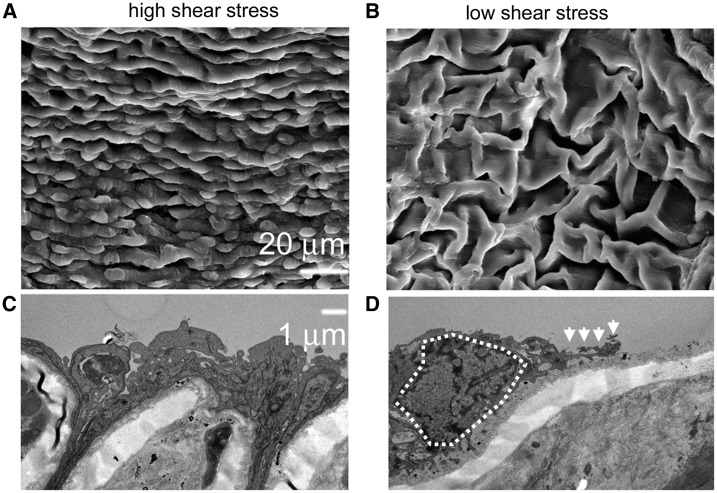
The ECs are normal in high shear stress region of the carotid. Electron micrographs of the ECs show the integrity or abnormality of the endothelium. (**A**) Typical features of normal ECs in high shear stress regions. (**B**) In low shear stress regions, overlapping of EC nuclei are seen, suggesting EC desquamation. (**C**) Typical features of normal ECs in high shear stress regions. (**D**) Typical feature of the initial stages of apoptosis. Chromatin condensation and margination can be seen in the dash line region of the downstream ECs. Furthermore, fragmentation of the nuclear and cell membranes is observed (arrows). Immunohistochemical staining demonstrates the effect of shear stress on eNOS expression

Intact endothelium covered the advanced atherosclerotic plaque in high shear stress regions (Fig. 6A). However, in low shear stress regions, the ECs were irregular in shape. The appearance of cuboidal phenotypes protruding into the lumen of the vessel and subendothelial surface was visible (Fig. 6B).

A typical feature of the initial stages of apoptosis, chromatin condensation and margination, can be seen in the ECs in low shear stress regions (Fig. 6D). Fragmentation of nuclear and cell membranes can also be seen in both of these micrographs. Some cells showed cell membrane blabbing, leading to the formation of apoptotic bodies. In contrast, the cells localized in the upstream direction were normal (Fig. 6C).

**Figure 7:**
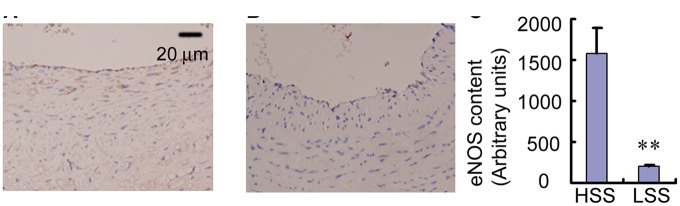
(**A**) eNOS expression in the upstream direction with high shear stress. (**B**) eNOS expression in the downstream with low shear stress (100× magnification). Black arrow represents cell denudation and no endothelium in some places in low shear stress regions. (**C**) Quantification of the expression of eNOS in the ECs. **P < 0.01, relative to the high shear stress regions

We further determined whether or not ECs in high shear stress regions had normal functions. eNOS is known to be a shear stress responsive gene that best characterizes the normal functions of ECs [27]. We analyzed the expression of eNOS by immunohistochemistry. High levels of eNOS expression occurred mainly in the endothelium in high shear stress regions (Fig. 7A), and almost no eNOS expression occurred in the endothelium in low shear stress regions (Fig. 7B). Denudation of the endothelium was also detected. The level of eNOS protein observed in arteries exposed to high shear stress was also signiﬁcantly higher compared with those exposed to low shear stress (1570.3 ± 345.5% versus 172.9 ± 49.9%, *P* < 0.01) (Fig.7C).

### High microvessels density and abnormal microvessels were in the high shear stress region

**Figure 8:**
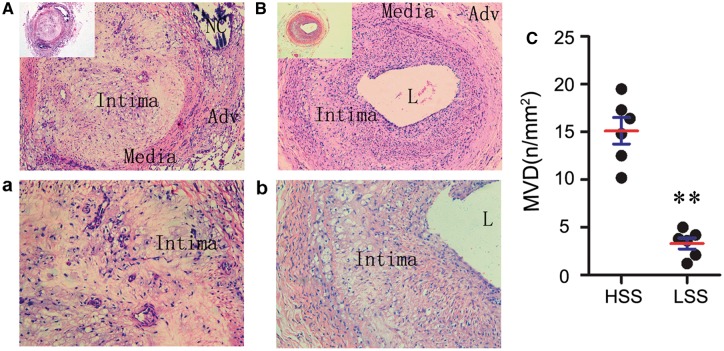
High density of the microvessel grows in the interplaque of the high shear stress region. Detail of hematoxylin and eosin–stained artery; inset shows entire artery in the upstream (**A**, **B**) and downstream (**C**, **D**) of the stenosis. Boxed regions c and d illustrate the region of interest showing microvessels in intraplaque region.(**E**)Mean microvessel density was quantified in the intraplaque region, and microvessel density (MVD) was higher in upstream of the stenosis. **P < 0.01, relative to the high shear stress regions

It is well-known that the number of the microvessels and the integrity of ECs in the plaque are critical for rupture-prone plaque formation [18]. As the statistic results in Fig. 8E, upon gross examination, microvessels were commonly observed in the intraplaque of high shear stress region (100%). There are a number of microvessels in intraplaque of high shear stress region (Fig. 8A), but less in low shear stress region (Fig. 8B). We counted the number of microvessels in the interplaque, results showed that the number of microvessels in the plaque is higher in the high shear stress region (Fig. 8C).

**Figure 9:**
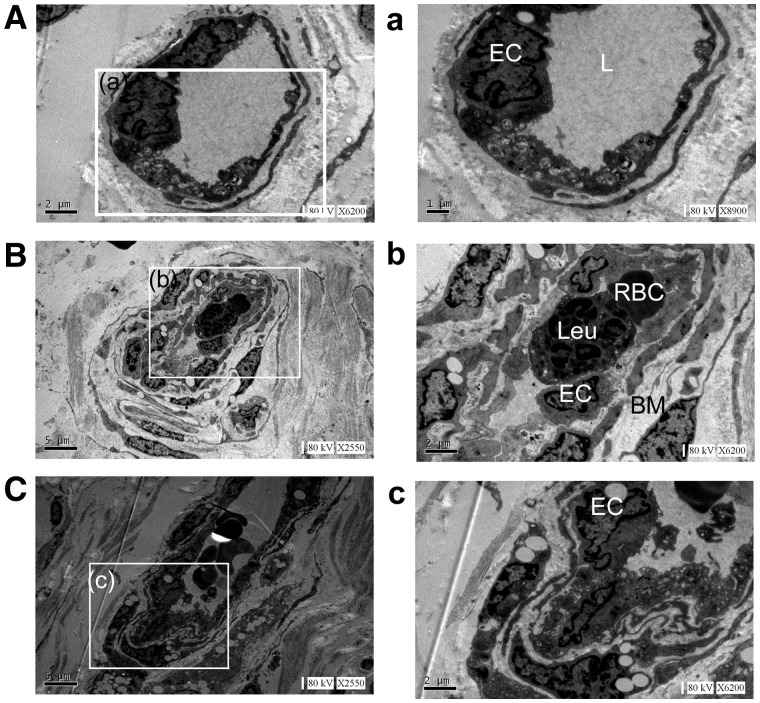
Most of ECs are abnormal in the plaque of high shear stress. Aberrant junctions, and leukocyte infiltration as shown by EM (**A**) Dysfunctional EC ultrastructure in an intraplaque microvessel: membrane blebs and intracytoplasmic vacuoles (white asterisk). (**B**) Ultrastructure of intraplaque microvessel with leukocytes (white asterisks). (b) Magnification of the boxed region in c showing aberrant inter-EC junction (white arrows) and basement membrane detachment (black arrow). (**C** and c) Leukocytes (white arrow) adhering to intraplaque microvessel endothelium

Then we analysed the endothelial integrity and interendothelial junctions by TEM. ECs of the microvessels in the intraplaque had blebbing and spike-like protrusions of the cell membrane, and the degradation of the basement membrane was also generally observed (Fig. 9A). And the cell membrane detachment was also observed (Fig. 9B). Based on the longitudinal section, we also detected the monocyte/macrophage infiltration in the tube (Fig. 9C).

## Discussion

When the atherosclerotic plaques intrude into the lumen of artery, the shear stress in the area surrounding the plaque changes substantially and the upstream region becomes the region of high shear stress [13]. Plaque rupture in the carotid artery is localized at the high shear stress region. Our current study builds the model that high shear stress induces integrity of the endothelium and the atherosclerotic rupture-prone plaque formation in rabbit carotid artery, and we also provide evidence that microvessels in the intraplaque are responsible for rupture-prone plaque formation in the high shear stress region.

VEGF is an angiogenic factor and can induce angiogenesis, and then induce atherosclerosis and plaque expansion [28]. The ECs in microvessels require mural cells to exert an adequate barrier function, and a chronic VEGF stimulus might be involved in the aberrant integrity of microvascular ECs and microvascular leakage [18]. High shear stress induces eNOS, and endothelial nitric oxide (eNO) mediated shear-induced angiogenesis in ECs [29, 30]. Thus, high shear stress induces the ECs to form tube-like structure and angiogenesis by increasing the synthesis of NO. But the high concentration of NO is also critical for the loss of smooth muscle cells and matrix [31]. Furthermore, ECs in microvessels require mural cells to exert an adequate barrier function the apoptosis of VSMC may be responsible for microvascular leakage [17]. And then this leaky vasculature is an entry point for inflammatory cells, red blood cells and lipid/lipoproteins. This may result in inflammation, intra-plaque haemorrhage, lipid core accumulation and eventually plaque rupture. In this paper, we proposed that angiogenesis disturb the balance which determines the stability of the caps of vulnerable plaques at upstream region of stenoses. This hypothesis suggests that angiogenesis is the reason why rupture-prone plaques are localized in the upstream region of plaques with high shear stress.

Currently, it is not clear that what reasons are mediated the angiogenesis by the high shear stress in the upstream of the stenosis. Previous work has suggested that high shear stress promotes the expression of angiogenesis-associated factors eNOS [19], and then release into the wall of the vessel and induce endothelial cell (EC) in the adventitia to form neovascularity.

Our hypothesis is that such a localization of vulnerable plaque on high shear stress region is due to the angiogenesis partly within the area. It may provide a new perspective for interpreting the location of plaque vulnerable to rupture and inhibiting plaque instability. Theoretical models could be developed to predict the relationship between the magnitude of shear stress and atherosclerosis plaque rupture. As a result, treatment modalities should be directed to reduce the amplitude of shear stress and angiogenesis. Also, heterogeneity in single plaques could reveal their stress-concentration points. The high endothelial shear stress regions may help us predict atherosclerosis-prone regions more precisely and direct surgical procedures to inhibit plaque development by controlling angiogenesis. It also could be applied to arterial bypass grafting and interventional procedures in which the geometry of the surgical intervention could be changed to the most appropriate geometry to adjust the shear stress for reducing the formation of neovascularization. Lastly, previous studies have shown that plaque neovascularization may serve as an interface for plaque expansion. So we can narrow the range of treatment strategy on our hypothesis that plaque angiogenesis is primarily localized on the upstream of plaque.

There are several limitations within the present study. A major limitation here is a limited number of time points were examined. However, we discovered that vulnerable plaques are difficult to form below 8 weeks. Second, the expression of eNOS mRNA and protein levels were not measured mainly because using complete segments of the vascular tissue for RNA determinations may mask variations caused by haemodynamic differences within the endothelium of the vascular segments. Third, the downstream of the stenosis has oscillatory flow maybe the reason that the blood flow in the upstream direction is high than the downstream.

In conclusion, increasing the number of angiogenesis or leaky vasculature induced by high shear stress may establish a more favorable microenvironment, which could diminish infiltration of inflammatory cells, deposition of lipoproteins and the occurrence of intra-plaque haemorrhage.

Competing financial interests. The authors declare no competing financial interests.
